# Midterm outcomes of electromagnetic computer-assisted navigation in minimally invasive total knee arthroplasty

**DOI:** 10.1186/1749-799X-8-37

**Published:** 2013-10-25

**Authors:** Satit Thiengwittayaporn, Auttakorn Kanjanapiboonwong, Detchart Junsee

**Affiliations:** 1 Department of Orthopaedics, Faculty of Medicine Vajira Hospital, Navamindradhiraj University, Bangkok 10300, Thailand; 2 Singburi Hospital, Amphur Muang, Singburi 16000, Thailand

**Keywords:** Minimally invasive total knee arthroplasty, Electromagnetic computer-assisted navigation, Outcomes, Midterm follow-up

## Abstract

**Background:**

A combination of two emerging technologies, computer-assisted navigation and minimally invasive surgery, in total knee arthroplasty has gained increasing interests from orthopedic surgeons around the world. To date, there has never been any midterm study for clinical and radiographic outcomes from using an electromagnetic computer-assisted navigation system. In this study, we aimed to systematically compare clinical and radiographic outcomes of minimally invasive surgery in total knee arthroplasty (MIS-TKA) performed with and without electromagnetic computer-assisted navigation at immediate and midterm follow-ups.

**Methods:**

A total of 151 patients (160 knees) who underwent MIS-TKA were randomized to be operated with electromagnetic computer-assisted navigation (group I: 75 patients, 80 knees) or without the navigation (group II: 76 patients, 80 knees). The clinical and radiographic outcomes of immediate, 6-week postoperative follow-up and average 6.1-year follow-up were compared.

**Results:**

On immediate, 6-week postoperative follow-up, clinical and radiographic outcomes did not reveal any difference between the two groups except for the fact that the operative time was longer in the navigation group. On 6.1-year follow-up, a total of 58 patients (63 knees) from group I and 58 patients (61 knees) from group II were reevaluated. There were no significant differences in clinical and radiographic loosening and in complications between the two groups.

**Conclusion:**

In this study, no significant differences of clinical and radiographic outcomes were found for immediate and midterm follow-ups of MIS-TKA performed with and without electromagnetic computer-assisted navigation except for the additional operating time in the navigation group.

## Background

A minimally invasive surgery (MIS) has been developed for total knee arthroplasty (TKA) in order to decrease early morbidity and improve patient outcomes. This technique utilizes small incision and down-sized instruments, which help in minimizing soft tissue dissection with no patellar eversion and no tibiofemoral joint dislocation [1-4]. Because of this small incision, minimally invasive surgery in total knee arthroplasty (MIS-TKA) may inevitably result in errors due to bone cutting and implant malpositioning. In the last few years, an emerging technique of computer-assisted navigation surgery (CAS) has therefore been developed to aid the surgeon in achieving better alignment in knee arthroplasty [5-7]. Together, these two new technologies are expected to improve short- and long-term outcomes of patients.

To date, several meta-analyses demonstrate that CAS-TKA provides a significant improvement in prosthesis alignment and component position. However, its clinical benefits are unclear and remain to be examined on a larger scale, randomized controlled trial with a long-term follow-up [8-10]. Although few studies reported a midterm result of CAS for knee arthroplasty, there has never been any midterm study for clinical and radiographic outcomes from using an electromagnetic computer-assisted navigation system. Therefore, in this study, we aimed to systematically compare clinical and radiographic outcomes of MIS-TKA performed with and without electromagnetic computer-assisted navigation on immediate and midterm follow-ups.

## Materials and methods

**Figure 1:**
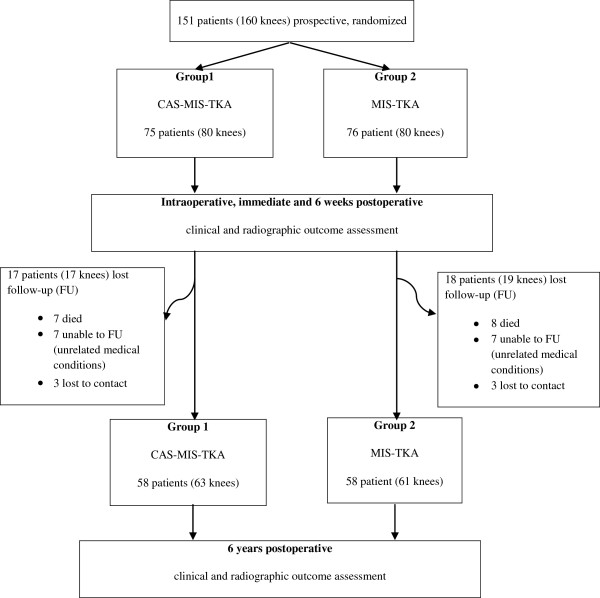
Consort chart to compare midterm outcomes of MIS-TKA with electromagnetic CAS-MIS-TKA and without the navigation system.

From January 2006 to January 2008, we conducted a prospective, randomized study in 151 patients (160 knees) who underwent MIS-TKA (Figure 1). Patients with knee osteoarthritis were included in this study if they had (1) symptoms that could not be improved by any nonoperative treatment, (2) a range of motion (ROM) that is more than 90°, (3) a flexion contracture that is less than 15°, and (4) no previous major knee operation. The average coronal deformity was 12.2° of anatomical varus (range, 12° of anatomical valgus to 32° of anatomical varus). An approval of this study was obtained from the Office of Vajira (Hospital) Institutional Review Board.

**Figure 2:**
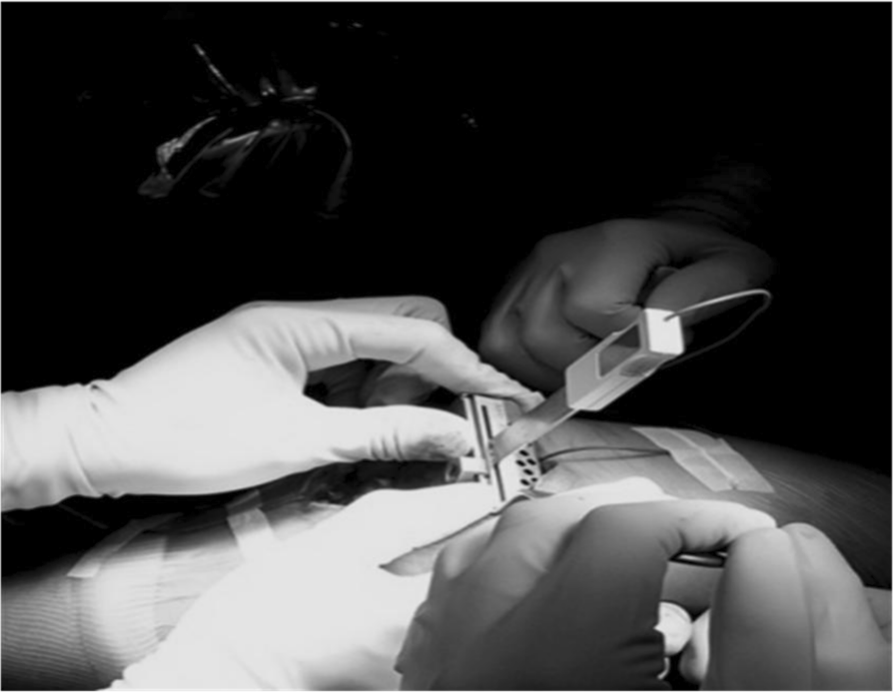
Use of electromagnetic CAS-MIS-TKA to guide the resection of the distal femur.

**Figure 3:**
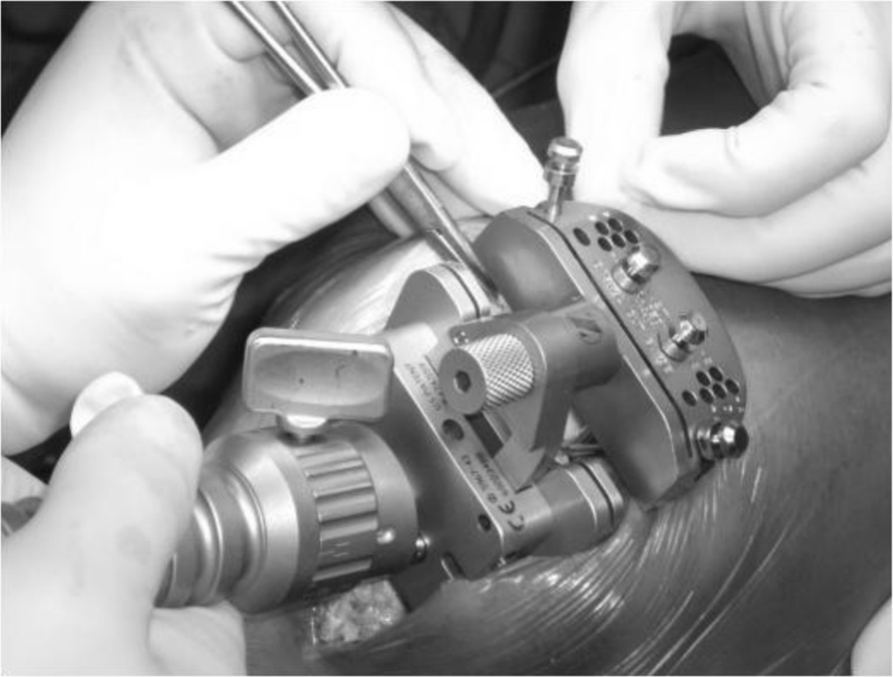
Minimally invasive total knee arthroplasty (MIS-TKA) using an intramedullary cutting guide to resect the distal femur.

A total of 151 patients (160 knees) who underwent MIS-TKA were randomized into two groups. Group I (CAS-MIS-TKA group; 75 patients, 80 knees) was operated using electromagnetic (Zimmer® Computer Assisted Solutions (CAS) Application: Electromagnetic Tracking Quad-Sparing™, Zimmer, Inc., Warsaw, IN, USA) computer-assisted minimally invasive technique (Figure 2). Group II (MIS-TKA group; 76 patients, 80 knees) was operated using the same minimally invasive technique but without the computer-assisted navigation (Figure 3). All patients were operated by the same surgeon.

All procedures were performed using the same fixed-bearing posterior stabilized implant (Nexgen HiFlex, Zimmer Inc., Warsaw, IN, USA) and using a minimally invasive technique. Patients had regional anesthesia unless contraindicated by a medical issue. The tourniquet pressure was at 280 mmHg in all cases. The incision was typically less than 9-cm long, which represented not more than twice the length of the patella. A mini-midvastus approach was employed, which allowed for an exposure of the knee without patellar eversion. CAS-MIS-TKA was performed using the electromagnetic computer-assisted navigation system. A femoral tracker was placed beneath the vastus medialis obliquus muscle at the midsagittal line about 4 cm above the joint, and a tibial tracker was placed on the medial tibial flare. A navigation system was used throughout the procedure to guide the bone cuts in both coronal and sagittal planes as well as to verify the final component position. For the MIS-TKA group, distal femoral resection was performed using the intramedullary technique and proximal tibial resection was performed by extramedullary technique. All components (femoral, tibial, and patellar) were cemented in all cases.

Both groups had the same postoperative pain control and rehabilitation consisting of a multimodal approach, which aims to avoid parenteral narcotics and early postoperative mobilization. Perioperative parameters (operative time and blood loss), clinical variables at 6 weeks post operation (range of motion, Knee Society scores (KSS)), and radiographic alignment at 6 weeks post operation were evaluated by two blinded, independent observers. Anteroposterior and lateral weight-bearing, long-leg radiographs at 6 weeks post operation were evaluated by measuring five component angles: (1) coronal tibiofemoral component angle (an angle between the femoral mechanical axis and the tibial mechanical axis as measured on the lateral side of the midline; neutral = 180°), (2) coronal femoral component angle (an angle between the mechanical axis of femur and the transcondylar line of the femoral component as measured on the lateral side; neutral = 90°), (3) coronal tibial component angle (an angle between the mechanical axis of the tibia and the tibial base plate as measured on the lateral side; neutral = 90°), (4) sagittal femoral component angle (an angle of femoral component flexion as measured on the posterior side of the midline; neutral = 90°), and (5) sagittal tibial component angle (a posterior slope angle of the tibial component as measured posteroinferiorly from the line perpendicular to the midline; neutral = 90°, with a 4° posterior slope as recommended by the manufacturer). Numbers of knees whose component angles were within ±3° from the neutral angle were considered to be acceptable outcomes as an alignment beyond this range predisposed to early implant failure [5,11-13]. All patients were scheduled for annual clinical and radiographic assessment. Any complications found during the study were recorded.

At an average of 6.1 years (range 5.2–7.3 years) post operation, a total of 58 patients (61 knees) from the MIS-TKA group and 58 patients (63 knees) from the CAS-MIS-TKA group were reevaluated for clinical and radiologic outcomes. Thus, 18 patients (19 knees) from the MIS-TKA group were lost to follow-up; of which, eight died from causes unrelated to the knees, seven were unable to attend the follow-up evaluation due to medical conditions unrelated to the knees, and three lost contact. For the CAS-MIS-TKA group, 17 patients (17 knees) were lost to follow up; of which, seven died from causes unrelated to the knees, seven were unable to attend the follow-up evaluation due to medical conditions unrelated to the knees, and three lost contact. Clinical radiographic outcomes and signs of component loosening were reevaluated according to the Knee Society Total Knee Arthroplasty Roentgenographic Evaluation and Scoring System [14]. Briefly, the width of the radiolucent lines was measured for each of three (femoral, tibial, and patellar) components. For the femoral component, seven zones on the lateral view were examined. For the tibial component, seven zones on the anteroposterior view and three zones on the lateral view were examined, whereas five zones on the Merchant view were examined for the patellar component. If the sum of the widths of radiolucencies in any component was greater than 10 mm, that component was considered loosening [14].

### Statistical analysis

Demographic and preoperative data of both groups were compared using independent *t* test (Student's *t* test) and chi square test. The clinical and radiographic outcomes of both groups were compared using an independent *t* test. The *p* value < 0.05 indicates a statistically significant difference (SPSS version 21, SPSS Inc, Chicago, IL, USA).

## Results

The CAS-MIS-TKA group consisted of 70 females and 5 males who had a mean age of 68.4 years (range 56–85 years). The MIS-TKA group consisted of 71 females and 5 males who had a mean age of 67.5 years (range 57–84 years). Other demographic variables such as body mass index and KSS [15] were similar between the two groups (Table 1).

**Table 1:** Demographic and preoperative data

	**MIS-TKA**	**CAS-MIS-TKA**	***P *** **value**
Age (year)	67 ± 7.68	68 ± 7.43	0.46
Body mass index	27.4 ± 3.89	26.7 ± 3.67	0.23
Male/female ratio	5:71	5:70	0.94
Number of comorbidities	1.4 ± 0.91	1.6 ± 1.18	0.18
ROM (deg)	111 ± 19.28	113 ± 16.28	0.64
Knee Society score			
Objective score	59 ± 11.74	60 ± 9.42	0.59
Functional score	40 ± 12.30	43 ± 9.27	0.18

An average value ± standard derivation is reported for each index. *ROM* range of motion.

### Perioperative and postoperative results at six weeks

Between the CAS-MIS-TKA and the MIS-TKA groups, most of the perioperative and 6-week postoperative clinical variables were not significantly different (*p* > 0.05), except for the significantly longer operative time in the CAS-MIS-TKA group (Table 2). Although blood loss was higher in the MIS-TKA group, the difference was not significantly different. Accordingly a combined average value between the objective score and functional score, the KSS for the MIS-TKA slightly improved from 99 to 151 and that of the CAS-MIS-TKA group also improved from 103 to 151 points.

**Table 2:** Perioperative and postoperative data at 6 weeks and 6 years

	**6 weeks**			**6 years**		
	**MIS-TKA**	**CAS-MIS-TKA**	**P value**	**MIS-TKA**	**CAS-MIS-TKA**	***P *** **value**
Operative time (min)	117 ± 21.77	159 ± 28.20	<0.001			
Blood loss (ml)	449 ± 238.75	423 ± 227.95	0.49			
ROM (deg)	110 ± 10.95	111 ± 10.03	0.69	121 ± 10.67	124 ± 8.34	0.16
Knee Society score						
Objective score	87 ± 4.26	86 ± 3.35	0.36	84.5 ± 3.55	85.0 ± 2.95	0.50
Functional score	64 ± 2.72	65 ± 2.31	0.49	66.7 ± 2.77	67.3 ± 4.64	0.36

An average value ± standard derivation is reported for each index. *ROM* range of motion.

To assess the accuracy of the operations in the two groups, at 6-week follow-up, deviation from the neutral knee angle was measured using angle deviation and percentage of knees with the implant aligned within 3° from the neutral angle as indicators by considering five angles (coronal tibiofemoral angle, coronal femoral component angle, coronal tibial component angle, sagittal femoral component angle, and sagittal tibial component angle; Table 3).

**Table 3:** Deviation outcomes from neutral angle

	**Angle deviation from neutral angle**			**Percentage of knees with implant aligned within ±3° from neutral angle**		
	**MIS-TKA**	**CAS-MIS-TKA**	***P *** **value**	**MIS-TKA (%)**	**CAS-MIS-TKA (%)**	***P *** **value**
Coronal tibiofemoral angle	0.8° varus	0.6° varus	0.61	82	91	0.16
Coronal femoral component angle	0.8° valgus	0.6° valgus	0.55	87	92	0.37
Coronal tibial component angle	0.9° varus	0.5° varus	0.25	95	92	0.44
Sagittal femoral component angle	2.9° flexion	2.6° flexion	0.08	77	85	0.26
Sagittal tibial component angle	4.8°	4.4°	0.17	80	91	0.09

They were assessed by angle deviation and percentage of knees with implant aligned within ±3° from the neutral angle.

### Postoperative clinical results at 6 years

A total of 58 patients (61 knees) from the MIS-TKA group and 58 patients (63 knees) from the CAS-MIS-TKA group were reevaluated for clinical and radiologic outcomes at, on average, 6.1 years post operation. Again, at the midterm postoperative follow-up, no significant difference was observed in terms of KSS and degrees of ROM between the two groups (Table 2).

### Complications

The following three complications were reported in the MIS-TKA group: one case of polyethylene exchange for postoperative recurvatum at 1 year post operation, one case with an open reduction and wiring for patellar fracture from direct trauma at 1 year post operation, and one case of readmission to the hospital from superficial skin infection. For the CAS-MIS-TKA group, there were four cases with complications: one case of open reduction and nailing for supracondylar fracture at an unrelated area from the position of tracker due to having fallen from the ladder at 2 years post operation, one case of manipulations under anesthesia for stiffness, one case of prolonged wound drainage, and one case of superficial skin infection. From these complications of both groups, it seemed that there were no obvious differences in incidence of complications (4 of 63 knees for the CAS-MIS-TKA group and 3 of 61 knees for the MIS-TKA group, *p* value = 0.73).

### 6-year postoperative radiographic results

There were no migrating or shifting prosthesis that should be considered as possible failure in both groups. There were 2 out of 61 knees in the MIS-TKA group and 3 out of 63 knees in the CAS-MIS-TKA group that exhibited <1-mm radiolucencies on the medial tibial plateau of the anteroposterior radiograph (zone 1 according to the Knee Society Total Knee Arthroplasty Roentgenographic Evaluation and Scoring System; *p* = 0.68) without clinical significance. For the femoral component, only 1 out of 61 knees in the MIS-TKA group and 2 out of 63 knees in the CAS-MIS-TKA group (*p* = 0.58) showed <1-mm radiolucencies on the posterior area of the lateral radiograph (zone 4) without clinical significance.

## Discussion

The combination of two emerging technologies, computer-assisted navigation and minimally invasive surgery in total knee arthroplasty, has gained increasing interests from orthopedic surgeons around the world. MIS-TKA has been reported to result in decreased pain, decreased blood loss, faster recovery, greater quadriceps muscle strength, improved cosmetic appearance, and higher patient satisfaction [2,16,17]. However, the small incision from MIS-TKA may also cause precision errors in bone cutting and implant malpositioning [2,17-19]. CAS therefore emerged to alleviate this potential pitfall of the MIS-TKA with the hope to improve the accuracy in knee arthroplasty alignment, especially with its limited exposure in the case of MIS.

Some meta-analysis reported that CAS could reduce the number of outliers in the limb mechanical axis and coronal position of the implant but could not demonstrate any short-term clinical difference [8-10]. Nevertheless, they expected that the more accurate alignment in CAS would provide a better long-term survival and clinical outcome. To date, only few studies have reported the midterm outcomes of CAS-TKA, and there were discrepancies in their conclusions. For instance, Hoppe et al. compared the clinical and radiographic outcomes of conventional, image-based and image-free TKA at a 5-year follow-up and concluded that the increased cost and time for CAS techniques did not result in a better medium-term outcome compared to the conventional technique [20]. Kamat et al. demonstrated that there was no difference in functional outcome between CAS-TKA and standard TKA at midterm follow-up [21]. However, Choong et al. compared the alignment, function, and patient's quality of life outcomes between patients undergoing standard TKA and CAS-TKA [22]. They found that CAS-TKA achieved greater accuracy in implant alignment, and the difference correlated with better knee function and improved quality of life. Therefore, this study was conducted to systematically compare the midterm outcomes between the MIS-TKA and the CAS-MIS-TKA.

There are two common types of imageless CAS surgery: the first one is infrared optical tracking system which requires a rigid bicortical fixation with pins, and the second one is electromagnetic tracking system which has a small tracker and needs only monocortical fixation [23]. One of the most frustrating problems with infrared optical trackers is the bone and soft tissue trauma [24]. The large, bicortical, multiple-hole violation of the bone has been implicated in fractures postoperatively. The smaller trackers of the electromagnetic tracking system resolve this problem. To the best of our knowledge, our study is the first to report on midterm outcomes of the electromagnetic tracking navigation system in MIS-TKA. Although our study has found that more knees aligned within ±3° in the CAS-MIS-TKA group than in the MIS-TKA group, the difference was not significant. Moreover, other clinical outcomes, radiographic loosening evaluation, and complications were also similar in both groups.

So far, we have not found any mechanical complications related to bone cutting error or component malpositioning in both groups. Moreover, no revision was required from any loosening problems for the duration of the study within the 6.1-year average. This midterm results in both groups show equally promising outcomes of MIS-TKA performed with or without electromagnetic computer-assisted navigation.

The primary shortcoming of this study is an inability to follow up the entire initial patient group at the midterm evaluation. However, the numbers of patients whom we were unable to follow up were approximately the same in both groups, resulting in an unbiased analysis of the comparison between the two groups in this study.

## Conclusions

We have not found any difference in the immediate and midterm clinical and radiographic outcomes between MIS-TKA and CAS-MIS-TKA using the electromagnetic tracking system. CAS-MIS-TKA, however, required significantly an additional operating time. The additional operating time must be considered by the surgeon to evaluate whether it is worth employing CAS without any significant improvement on the patients' midterm outcomes. To serve the orthopedic community further, a similar assessment of long-term outcomes should be conducted.

## Competing interests

The authors declare that they have no competing interests.

## Authors' contributions

ST conceived the idea of this study, prepared the protocol, collected data, and wrote the manuscript. AK collected and analyzed the data. DJ participated in the preparation of the protocol, data collection, and analysis of data. All authors read and approved the final manuscript.
